# Morphological and Immunophenotypical Changes of Human Bone Marrow Adipocytes in Marrow Metastasis and Myelofibrosis

**DOI:** 10.3389/fendo.2022.882379

**Published:** 2022-06-08

**Authors:** Michele Dello Spedale Venti, Biagio Palmisano, Samantha Donsante, Giorgia Farinacci, Flavia Adotti, Ilenia Coletta, Marta Serafini, Alessandro Corsi, Mara Riminucci

**Affiliations:** ^1^ Department of Molecular Medicine, Sapienza University of Rome, Rome, Italy; ^2^ Centro Ricerca M. Tettamanti, Department of Pediatrics, University of Milano-Bicocca, Monza, Italy

**Keywords:** bone marrow, marrow metastasis, myeloproliferative neoplasia, myelofibrosis, bone marrow adipose tissue, bone marrow adipocytes, histomorphometry, immunohistochemistry

## Abstract

The bone marrow adipose tissue constitutes more than two-thirds of the bone marrow volume in adult life and is known to have unique metabolic and functional properties. In neoplastic disorders, bone marrow adipocytes (BMAds) contribute to create a favorable microenvironment to survival and proliferation of cancer cells. Many studies explored the molecular crosstalk between BMAds and neoplastic cells, predominantly in *ex-vivo* experimental systems or in animal models. However, little is known on the features of BMAds in the human neoplastic marrow. The aim of our study was to analyze the *in situ* changes in morphology and immunophenotype of BMAds in two different types of neoplastic marrow conditions. We selected a series of archival iliac crest and vertebral bone biopsies from patients with bone marrow metastasis (MET), patients with myeloproliferative neoplasia with grade-3 myelofibrosis (MPN-MF) and age-matched controls (CTR). We observed a significant reduction in the number of BMAds in MET and MPN-MF compared to CTR. Accordingly, in the same groups, we also detected a significant reduction in the mean cell diameter and area. Immunolocalization of different adipocyte markers showed that, compared to CTR, in both MET and MPN-MF the percentages of adiponectin- and phosphorylated hormone sensitive lipase-positive BMAds were significantly reduced and increased respectively. No statistically significant difference was found between MET and MPN-MF. Interestingly, in one MET sample, “remodeled” BMAds containing a large lipid vacuole and multiple, smaller and polarized lipid droplets were identified. In conclusion, our data show that in different types of marrow cancers, BMAds undergo significant quantitative and qualitative changes, which need to be further investigated in future studies.

## Introduction

The bone marrow adipose tissue (BMAT) (1) constitutes more than two-thirds of the bone marrow (BM) volume in adult life (2, 3). It is comprised of cells, BM adipocytes (BMAds), that bear a single, large cytoplasmic lipid droplet and express adipose-lineage markers such as fatty acid binding proteins, leptin, adiponectin and others (4). BMAds appear at birth in the extravascular marrow space to contribute to the three-dimensional stromal cell network that holds hematopoietic cells, and progressively expand during skeletal growth in parallel with the centripetal retraction of the hematopoiesis (Neumann’s law) (5). Based on this inverse relationship with the hematopoietic tissue, BMAds have long been thought as white fat cells with a simple supporting/filling function. However, it is now widely recognized that BMAds represent a distinct fat depot with metabolic and functional properties that are different from those of the other human fat tissues (6). In addition, it is now well known that BMAds participate in the regulation of skeletal homeostasis, in systemic metabolism and in the pathogenesis of different bone and BM diseases (4, 7).

Over the last years, the potential pathogenetic role of BMAds and its molecular bases have been intensively investigated in marrow neoplastic conditions such as hematological malignancies and cancer metastasis. Thus, it has been shown that BMAds are able to modulate the migration and aggressiveness of neoplastic cells (8), to sustain their growth (9) and, in some cases, to promote their survival during therapy (10, 11). These effects are achieved by multiple mechanisms such as the generation and transfer of fatty acids (9) and the secretion of different types of adipokines (4). On the other side, it has also been reported that tumor cells are able to harness the metabolic and biological functions of BMAds to generate a microenvironment that promotes their own growth. The tumor-dependent modifications of BMAds reported so far include the stimulation of a lipolytic state (9), the increased expression of lipid transporter genes (9) and the induction of a senescence-associated secretory phenotype (12). However, although these studies have significantly expanded the knowledge on the molecular crosstalk between BMAds and neoplastic cells, they have been performed predominantly in *ex-vivo* experimental systems or in animal models thus providing little, if any, information on the *in situ* features of BMAds in the human neoplastic BM.

In this study, we have analyzed the number, morphology and phenotype of BMAds in archival bone biopsies obtained from patients with marrow metastasis, either from epithelial and non-epithelial tumors, and myeloproliferative neoplasms featuring grade-3 myelofibrosis. We selected BM metastasis since *in situ* features of BMAds in this condition have never been investigated. The samples of chronic myeloproliferative neoplasms were chosen based on the absence of data on the *in situ* immunophenotype of BMAds in these haematological malignancies. In order to generate a homogenous experimental group, the latter samples were selected based on the presence of myelofibrosis, which is frequently found in patients with chronic myeloproliferative neoplasms. We report that BMAds in the neoplastic marrow samples were characterized by altered histomorphometric and immunophenotypic features that might reflect their tumor-promoting activity.

## Materials and Methods

### Bone Biopsies

Human trephine BM biopsies with either BM metastasis (MET) or myeloproliferative neoplasms with grade-3 myelofibrosis (MPN-MF) performed between 2012 and 2020 were retrospectively identified in the archive of the Section of Pathology of the Policlinico Umberto I Hospital of Rome, Italy.

The MET group was composed of 8 patients (7 females and 1 male, median age 55 years, range 27-73 years), affected by breast carcinoma (four patients, two with infiltrating ductal carcinoma, one with infiltrating lobular carcinoma and one undefined), signet-ring cell carcinoma of the stomach (one patient), high-grade neuroendocrine carcinoma and undifferentiated carcinoma of unknown origin (one patient each) and malignant glioma (one patient). In the last patient the sample was obtained from vertebral BM. The quantitative data obtained from the analysis of this sample have been considered within the MET group as iliac crest and vertebrae are both axial hematopoietic (red) marrow sites (5). The MPN-MF group included 8 patients (6 females and 2 males, median age of 58.5 years, range 39-81 years). Iliac crest biopsies performed for lymphoproliferative disease staging in the same period of time in eight patients (6 females and 2 males, median age 52.5 years, range 41-68 years) with no evidence of BM involvement by either neoplasia or other pathologies were also selected in the same archive and used as control (CTR) group. The study was conducted according to the guidelines of the Declaration of Helsinki and approved by the Institutional Review Board (Department of Molecular Medicine, Sapienza University of Rome, Italy, 17/02/2022). Written informed consent was not deemed necessary owing to the retrospective nature of the study.

### Histology and Immunohistochemistry

Biopsies were fixed in 4% phosphate-buffered formaldehyde and routinely processed for paraffin embedding after decalcification with ethylenediaminetetraacetic acid in disodium salt acid buffer (Osteodec, Bio-Optica, Milan, Italy). For diagnostic purpose, the slides were examined by two of the authors. For this study, 4µm thick tissue sections were re-cut from each paraffin block and used for haematoxylin-eosin (HE) stain and for immunohistochemistry. Immunohistochemical analysis was performed in a blinded fashion as described previously (13, 14). The following primary antibodies diluted in phosphate buffer were used: polyclonal anti-human perilipin-1 (PLIN1) (Abcam, Cambridge, UK, ab3526, 1:500); polyclonal anti-human fatty acid binding protein 4 (FABP4) (Abcam, ab13979, 1:200); monoclonal anti-human adiponectin (ADIPOQ) (InVitrogen, Carlsbad, CA, USA, MA1-054, 1:50); polyclonal anti-human phosphorylated-hormone sensitive lipase antibody (p-HSL) (InVitrogen, PA5-38087, 1:100). Primary antibodies were omitted in negative technical controls. The color reaction was developed using 3,3’-diaminobenzidine tetrahydrochloride (DAB, Sigma, St- Louis, MO) as substrate.

### Histomorphometry

Histomorphometric analysis was performed in a blinded fashion and according to the guidelines of the Bone Marrow Adiposity Society (1, 15). HE-stained and immunostained sections were scanned *via* Aperio Scan Scope CS (Leica Biosystem Imaging, Nußloch, Baden-Wurttemberg, Germany) and analyzed using ImageJ (16). For each scanned section, 10 snapshots were taken at 20x magnification from the central portion of the trephine biopsies. The region of interest (ROI) ranged from 1.35 mm^2^ to 1.93 mm^2^. Bone trabeculae, areas of hemorrhages, harvesting or processing tissue damage artifacts were excluded. Marrow area (Ma.Ar), number of BMAds per marrow area (N.Ad/Ma.Ar), maximum diameter (Ad.Dm) and area (Ad.Ar) of individual BMAds were measured on HE-stained sections. Immunostained pictures were used to calculate the fraction of immunoreactive BMAds on the total number of adipocytes by ImageJ plugin Cell Counter.

### Statistical Analysis

Ordinary one-way ANOVA test with Tukey’s multiple comparison test was performed by GraphPad Prism 8.0.2 software. A *p*-value less than 0.05 was considered statistically significant.

## Results

### BMAd Number and Size Reduction in Marrow Metastasis and Myeloproliferative Neoplasia

**Figure 1:**
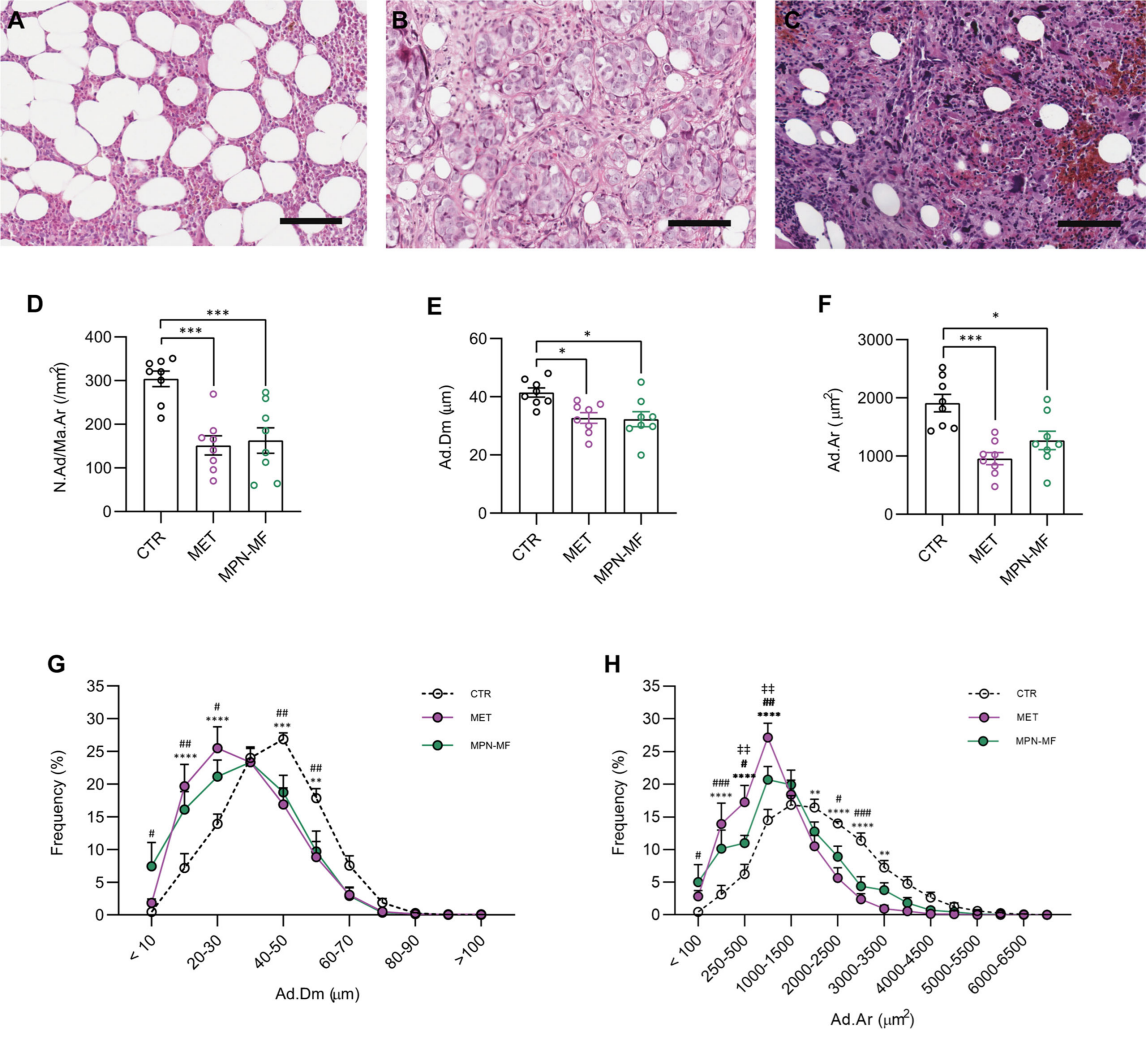
Representative histologic images of CTR **(A)**, MET **(B)** and MPN-MF **(C)** bone biopsies. Histomorphometric analysis of BMAds number (**D**, N.Ad/Ma.Ar), diameter (**E**, Ad.Dm) and area (**F,** Ad.Ar). Frequency distribution curves of BMAds diameter and area are illustrated in **(G**, **H)**, respectively. Scale bar: 100 µm. In **(D**–**F)**: *p < 0.05, ***p < 0.001; In **(G**, **H)**: CTR *vs* MET: **p < 0.01, ***p < 0.001, ****p < 0.0001; CTR *vs* MPN-MF: ^#^ *p* < 0.05 and ^##^ *p* < 0.01, ^###^ *p* < 0.001; MET *vs* MPN-MF: ^‡‡^ *p* < 0.01 .

The microscopic review of all bone biopsies confirmed the presence of normal haematopoiesis in the CTR group (**Figure 1A**); non-hematological neoplastic cells were observed in the MET group (**Figure 1B**), while atypical hematopoietic cells separated by fibrous bundles were present in the MPN-MF biopsies (**Figure 1C**).

The comparative analysis of the adipocyte number in the different groups revealed a significant reduction in the two neoplastic conditions compared to the control biopsies (CTR vs MET *p* = 0.0004; CTR vs MPN-MF *p* = 0.001) (**Figure 1D**). Moreover, histomorphometric analysis demonstrated a statistically significant reduction in the adipocyte diameter (CTR vs MET *p* = 0.017 and CTR vs MPN-MF *p* = 0.013) and area (CTR vs MET *p* = 0.0003, CTR vs MPN-MF *p* = 0.0104) in the neoplastic samples compared to the controls (**Figures 1E, F**). In contrast, no difference between the two neoplastic conditions was observed for adipocyte number, diameter and area (**Figures 1D** **–G**), although the frequency distribution of adipocyte area revealed a higher frequency of smaller adipocytes in the MET group (**Figures 1B, H**). These data indicate that marrow metastasis, from epithelial and non-epithelial tumors, and myeloproliferative neoplasms featuring grade-3 myelofibrosis, are associated with a significant reduction in the number of adipocytes and with an increase in small adipocytes.

### BMAd Immunophenotypic Changes in Marrow Metastasis and Myeloproliferative Neoplasia

We performed immunohistochemistry to compare the fractions of BMAds marked by the presence of PLIN1, FABP4, ADIPOQ and p-HSL in the different experimental groups.

**Figure 2:**
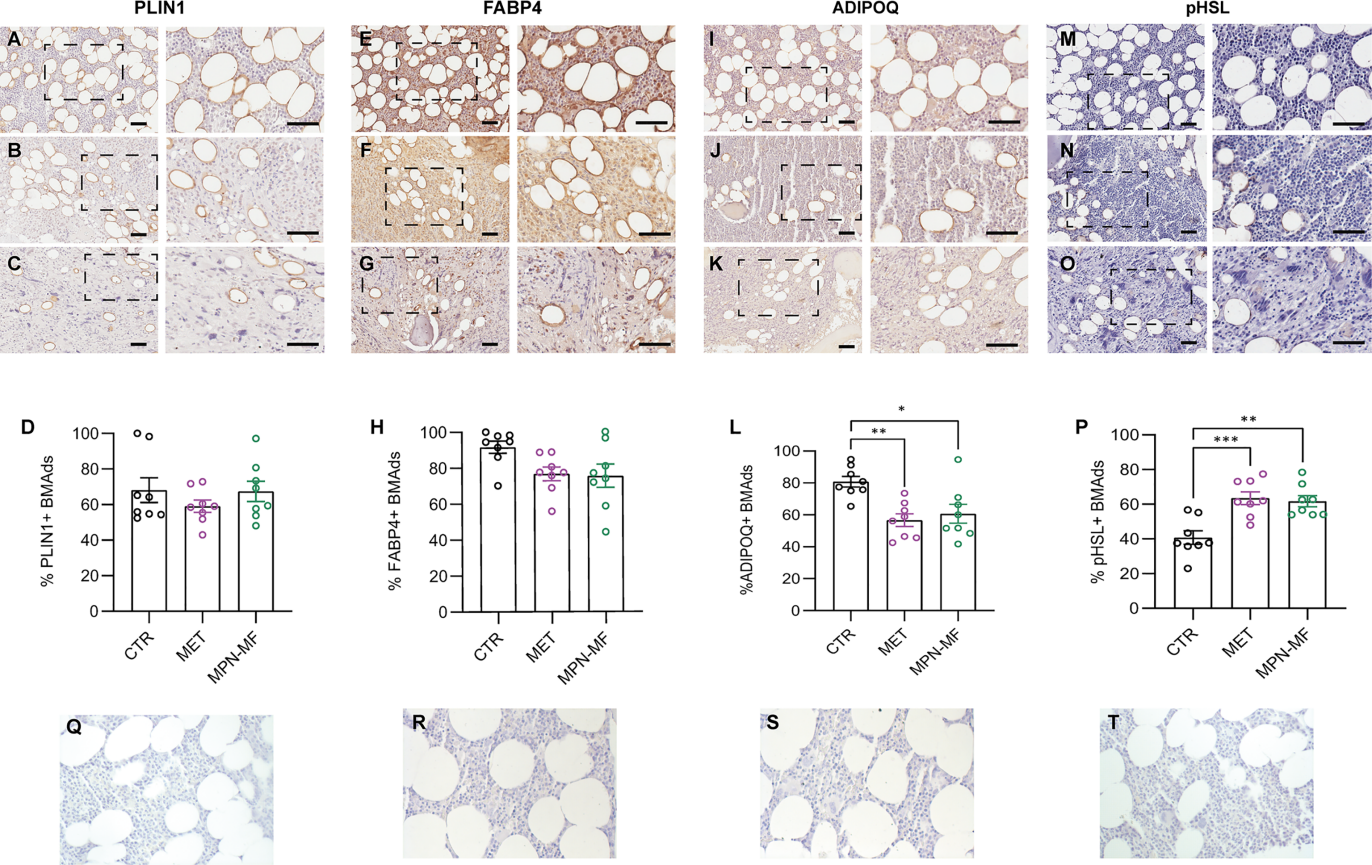
Representative images of PLIN1-, FABP4-, ADIPOQ- and p-HSL-immunostained sections from CTR **(A, E, I, M)**, MET **(B, F, J, N)** and MPN-MF **(C**, **G**, **K**, **O)** bone biopsies. The right panels of each image illustrate the zoomed boxed area. Scale bars: 50 µm. Graphs in **(D**, **H**, **L**, **P)** represent the quantitative analysis of each immunostaining. Negative technical controls in which primary antibodies were omitted are shown in panels **(Q–T)**. *p < 0.05, **p < 0.01; ***p < 0.001.

The percentage of PLIN1-positive BMAds was not significantly different among the three groups (**Figures 2A** **–D, Q**). Similarly, the percentage of FABP4-positive BMAds was not significantly different among the three groups even though a trend towards reduction was observed in the neoplastic samples (CTR vs MET *p* = 0.0903; CTR vs MPN-MF *p* = 0.0638) (**Figures 2** **E–H, R**).

Interestingly, the number of BMAds expressing ADIPOQ was significantly reduced both in MET (*p* = 0.0032) and MPN-MF (*p* = 0.0136) compared to CTR (**Figures 2I** **–L, S**). Since AdipoQ has been reported to enhance adipocyte lipid storage (17, 18), this result may suggest a reduction of this process in BM involved by neoplasia. pHSL immunostaining revealed a higher number of positive adipocytes in neoplastic biopsies (**Figures 2M** **–P, T**), in which their number was higher by almost 50% compared to CTR (**Figure 2P**, CTR vs MET *p* = 0.0006; CTR vs MPN-MF *p* = 0.0014). No significant difference in the percentage of positive BMAds was detected between the two types of tumors (ADIPOQ: MET vs MPN-MF *p* = 0.8087; pHSL: MET vs MPN-MF *p* = 0.9373) (**Figures 2L, P**).

**Figure 3:**
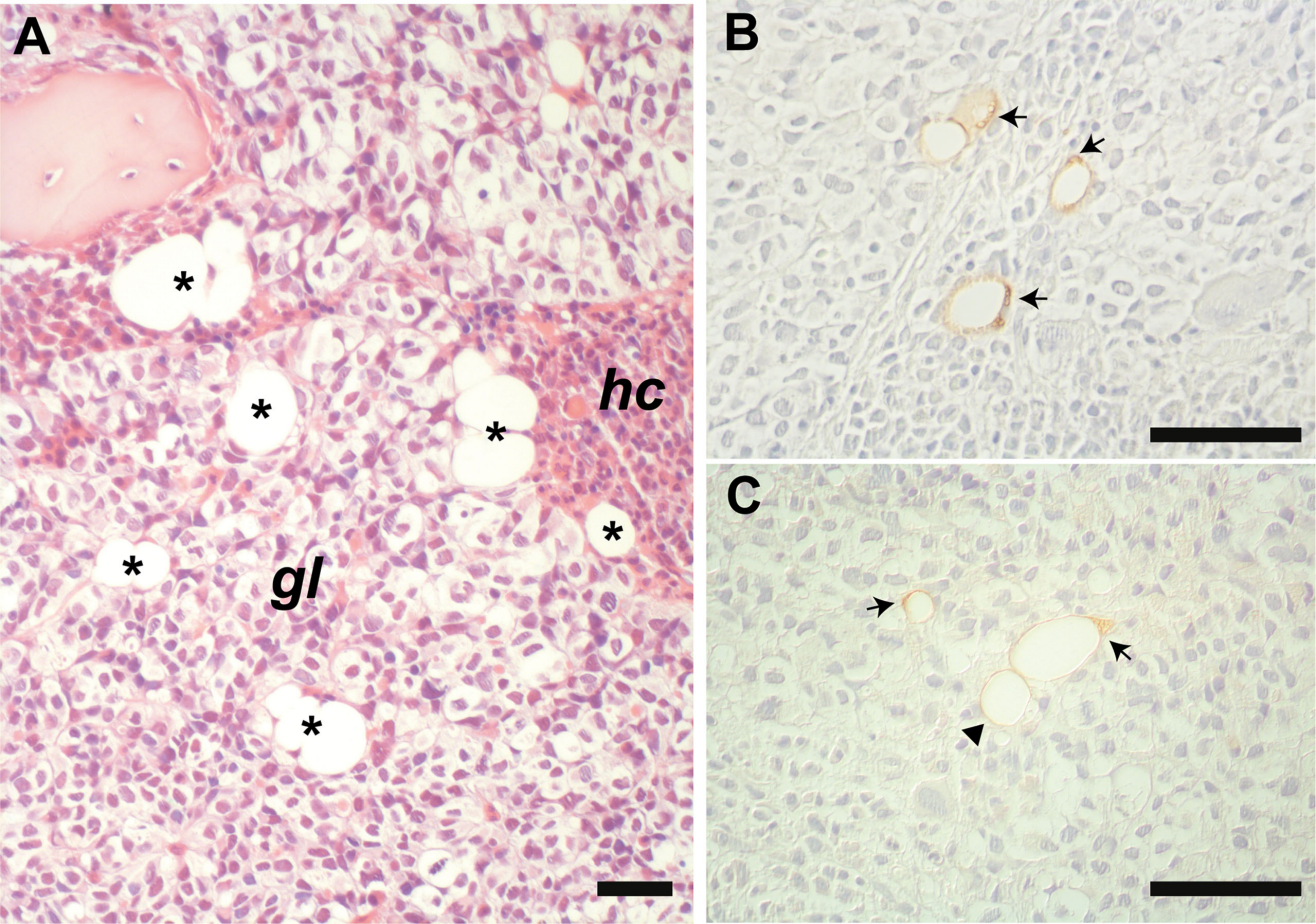
Representative histological image of the marrow metastasis of glioma **(A)**. BMAds (asterisks) are intercalated among the neoplastic cells (*gl*) and the haematopoietic cells (*hc*). Immunostaining for PLIN1 **(B)** and ADIPOQ **(C)** highlights intra-tumoral “remodeled” BMAds containing very small intracytoplasmic lipid droplets clustered together and polarized beneath the plasma membrane (arrows), which are not observed in morphologically typical unilocular adipocyte (arrowhead). Panel **(A)**: HE stain. Scale bars: 50 µm.

Interestingly, in the bone biopsy from the patient with metastasis of malignant glioma (**Figure 3A**), we observed an unusual morphology of BMAds characterized by the presence of a large vacuole associated with small lipid droplets that were clustered together beneath the plasma membrane, as better shown by ADIPOQ **Figure 3B**) and PLIN1 (**Figure 3C**) immunostaining.

## Discussion

The human BM is the site of origin of most haematological malignancies and a frequent site of metastasis of non-haematological tumors. In marrow neoplasia, the BMAT undergoes metabolic and molecular changes that help cancer cells to thrive at the expense of the other BM cell types. BMAds are known to secrete adipokines (e.g., adiponectin and leptin) and inflammatory factors (e.g., interleukin-6 and tumor necrosis factor-α) and are thought to be the energy source of tumor cells (e.g., through the release of free fatty acids), which, in turn, modulate their activities by releasing different types of molecules (e.g., cytokines and chemokines) (4, 8, 11, 19).

Previous work showed that in peripheral white adipose tissue (WAT), adipocytes located in close proximity of invasive cancer cells undergo morphological changes [i.e., loss of lipid content (delipidation) and acquisition of a fibroblast-like/pre-adipocyte phenotype (dedifferentiation)] and functional modifications (e.g., decreased expression of adipocyte-related genes and increased production of pro-inflammatory cytokines) (20). However, only a few studies investigated the *in situ* morphology and phenotype of BMAT in the human neoplastic BM.

The analysis of BMAds neighboring marrow tumors may provide information of relevance for the pathogenesis and possibly for the diagnosis of marrow neoplastic diseases. For example, it may reveal changes of BMAds that occur at a specific time (e.g., early *vs* late stage of marrow cancer infiltration) and/or anatomical space (e.g., in subcortical *vs* perisinusoidal BMAT) during the progression of the disease. On the other side, it may assist in the diagnostic identification of neoplastic BM samples when cancer cells are very few and barely detectable. Currently available work on the *in situ* features of BMAds in the neoplastic BM consists essentially in a few histomorphometry studies performed exclusively on hematological tumors (21).

Here, we report the results of the quantitative and qualitative analyses of BMAds in bone biopsies from patients with different types of marrow cancer metastasis and with myeloproliferative neoplasia with grade-3 myelofibrosis. We provide the first data on the *in situ* immunophenotype of BMAds in haematological malignancies and the first analysis ever of their *in situ* quantitative and immunophenotypic features in BM cancer metastasis.

Our study revealed a significant decrease in the number of BMAds in affected bone biopsies, regardless of the type of neoplasia. The quantitative reduction of BMAds in the presence of tumors seems to be a recurrent finding and was previously observed in Multiple Myeloma (MM) (12), Acute Lymphoblastic Leukaemia (ALL) (22), Myelodysplasia (21, 23), Acute Myeloid Leukaemia (AML) (24) and MPN (21). The reason for the lowering of BMAds in marrow neoplastic conditions is currently under investigation. Some studies showed that it is unrelated to the amount of marrow cellularity and may not be explained solely by the physical pressure caused by tumor infiltration (12, 22). Other studies reported a compromised growth and differentiation of BMAd progenitor cells (22, 24, 25). BMAds derive from skeletal stem/progenitor cells that appear after birth in the marrow cavity (26) and are included within the fibroblast-like cell subset of the BM stroma (Bone marrow stromal cells, BMSCs) (27). Reduced adipogenic differentiation was shown in BMSCs isolated from AML patients, in which the defective generation of mature adipocytes was not reproduced in the extra-skeletal WAT depots (24), and from patients with MM (25). In addition, in ALL patients, Heydt et al. observed an altered growth of BMSCs with accumulation of adipocyte-primed cells and suggested that the differentiation of adipocyte progenitors was stalled by blast-dependent mechanisms (22). Interestingly, we previously observed that BM stromal progenitor cells isolated from patients with AML were characterized by an enhanced terminal adipogenic differentiation compared to controls when transplanted *in vivo* in the absence of leukemic blasts (28). The negative modulation of BM adipogenesis seems to be in contrast with the positive effect of BMAds on cancer growth. A plausible explanation for our results is that this positive effect is an early event no longer required in the established disease. However, it must be reminded that BMAds also support the maturation of the normal myeloid-erythroid lineages (24), which compete with the neoplastic cells for the same microenvironment. Thus, it is possible that the modulation of BMAd number in the neoplastic marrow reflects, at least in part, the need for the tumor to generate a supporting microenvironment while suppressing the normal haematopoiesis.

We also observed a reduced size of BMAds in all our samples, consistent with previous report on MM (29) and other lympho- and myelo-proliferative conditions (21). The change in the dimension of individual BMAds was previously related to the enhanced lipolysis induced by neoplastic cells (9, 30), although subsequent studies did not detect a lipolytic activity in BMAds (6). Regardless of the mechanism(s), the shrinking of BMAds may have multiple potential consequences on the neoplastic microenvironment that should not be overlooked. For example, since BMAds are in close contact with the marrow sinusoidal network (31), a pronounced modification of their dimension may affect the blood flow and, as a consequence, the amount of oxygen and nutrients for neoplastic cells. In addition, as previously reported in WAT, variations in the cell size within BMAT could be correlated with different chemokine and lipid secretory profiles and therefore with different modulatory effects on the tumor growth (32).

As major changes in the overall phenotype of BMAT, we detected a reduced number of ADIPOQ positive and an increased number of p-HSL positive BMAds in the neoplastic marrow compared to control biopsies. ADIPOQ is the most abundant molecule produced by the adipose tissue and in humans it is highly expressed in BMAT compared to WAT (33). Reduced levels of ADIPOQ, in parallel with an increase in the level of leptin, are associated with an enhanced risk of development of different tumors, including hematological neoplasia (34, 35). Indeed, ADIPOQ inhibits proliferation and promotes apoptosis in different types of cancer cells (36). Recently, it has been reported that the content of this adipokine in the marrow plasma is lower in patients with ALL compared to healthy subjects (22). In agreement with these data, we demonstrate here for the first time a reduction in the number of ADIPOQ positive BMAds in the presence of cancer metastasis and myeloproliferative neoplasia with grade-3 myelofibrosis, thus confirming that the modulation of the secretory activity of BMAT contributes to the changes in the marrow/serum levels of this adipokine in neoplastic conditions.

HSL is a lipase that is activated upon phosphorylation and is involved in the breakdown of triglycerides (37). Cancer cells may upregulate its expression and phosphorylation in WAT (38, 39). Although BMAds seem to have a specific lipid metabolism compared to WAT (6), HSL phosphorylation was reported to be induced in BMAds co-cultured with ALL blasts (9). In this study we have confirmed this effect *in situ* showing that, in the presence of neoplastic cells, the fraction of p-HSL positive BMAds was expanded. The metabolic implication of the increase of p-HSL and its significance in the context of BM cancer involvement remains to be clarified. However, it is interesting to note that in the marrow metastasis of glioma, we observed BMAds bearing a large lipid vacuole and a polarized cluster of small droplets beneath the plasma membrane. This finding is reminiscent of the remodeled BMAds previously detected in the mouse femur upon stimulation with a β3 adrenergic receptor agonist (40). Thus, it is possible that a remodeling phenomenon occurs in human BMAds too, at least in some specific rare conditions as the exposure to glioma cells. Of note, it is known in the literature that astrocytomas may cause lipodystrophy (41) and that lipolysis is increased in the glioma microenvironment (42).

In conclusion, although this study has important limitations (such as the reduced number of bone biopsies, the absence of correlation with patients’ clinic and anthropometric data), it demonstrates for the first time that, in different types of marrow cancers, BMAds undergo significant quantitative and morpho-phenotypical changes, which need to be further investigated in future studies.

## Data Availability Statement

The raw data supporting the conclusions of this article will be made available by the authors, without undue reservation.

## Ethics Statement

The studies involving human participants were reviewed and approved by Institutional Review Board, Department of Molecular Medicine, Sapienza University of Rome, Italy, 17/02/2022. Written informed consent was not deemed necessary owing to the retrospective nature of the study.

## Author Contributions

MDSV, BP, SD, GF, FA and IC performed morphometric analysis and immunohistochemical stains. MDSV, BP, MS, AC and MR analyzed the data. MDSV and BP performed statistical analysis and wrote the draft of the manuscript. MDSV, BP, MS, AC and MR edited the manuscript. All authors approved the final version of the manuscript.

## Funding

Sapienza University to AC (RM118164289636F0) and MR (RM11916B839074A8 and RM120172B8BF5C15).

## Conflict of Interest

The authors declare that the research was conducted in the absence of any commercial or financial relationships that could be construed as a potential conflict of interest.

## Publisher’s Note

All claims expressed in this article are solely those of the authors and do not necessarily represent those of their affiliated organizations, or those of the publisher, the editors and the reviewers. Any product that may be evaluated in this article, or claim that may be made by its manufacturer, is not guaranteed or endorsed by the publisher.
